# Supplementary figures and images for: Tau, XMAP215/Msps and Eb1 co-operate interdependently to regulate microtubule polymerisation and bundle formation in axons
Source: PLoS Genet. 2021 Jul 6;17(7):e1009647. doi: 10.1371/journal.pgen.1009647 (PMC8284659; doi:10.1371/journal.pgen.1009647)

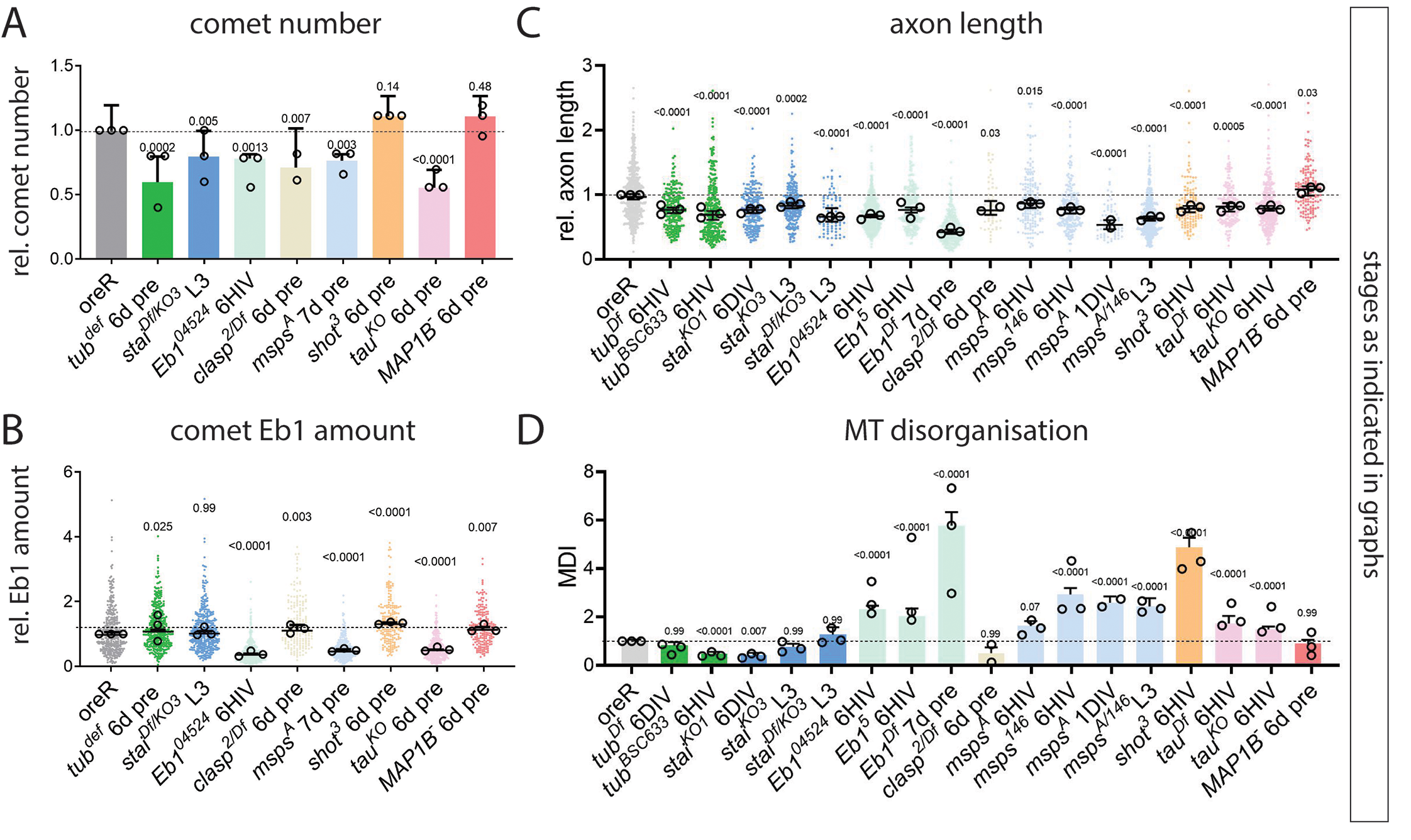

Supplement: S1 Fig — Graphs show extended data sets for four of the parameters displayed in Fig 1 (indicated above each graph). Data points/bars representing mutant conditions for different genes are consistently colour-coded in all graphs, and conditions used are indicated below (6HIV, cultured from embryos for 6hrs; 6d/7d pre, cultured from embryos for 12hrs following 6 or 7 days pre-culture; L3, cultured from late larval CNS for 18hrs). Allele names are given as superscript: absence of slash indicates homozygous, presence of slash hetero-allelic conditions. Data were normalised to parallel controls (dashed horizontal line) and are shown as median ± 95% confidence interval (B,C) or mean ± SEM (A,D); data points from at least two experimental repeats consisting of 3 replicates each are shown, large open circles in graphs indicate median/mean of independent biological repeats. P-values obtained with Kruskall-Wallis ANOVA test above data points/bars. For raw data see S7 Data. (TIF) [file pgen.1009647.s001.tif]

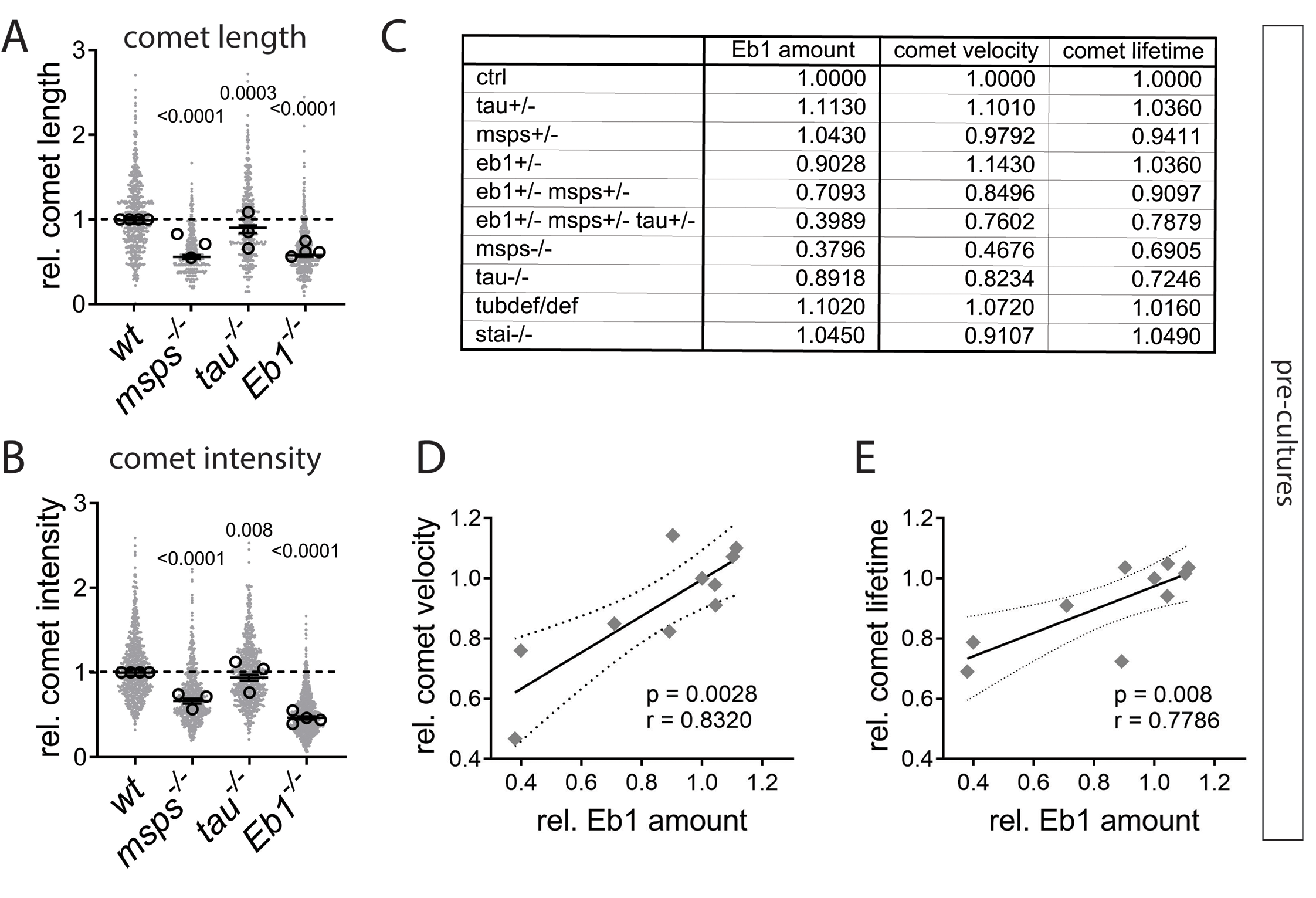

Supplement: S2 Fig — A,B) Eb1 amount at comets is calculated as the product of comet length (A) and the fluorescent mean intensity of Eb1 comets (B), which are both affected to similar degrees by homozygous condition of mspsA, tauKO and Eb104524 in embryo-derived neurons cultured for 12hrs following 6 day pre-culture (6d pre); data were normalised to controls (dashed horizontal line) and are shown as scatter dot plot with median ± 95% confidence interval of at least three experimental repeats, large open circles in graphs indicate median/mean of independent biological repeats. P-values listed above each plot were obtained with Kruskall-Wallis ANOVA tests. C) The table lists data for Eb1 amounts (fixed neurons; compare Fig 1A–D and 1J) or for comet velocity/lifetime (live imaging; compare Fig 1K and 1L), all obtained from pre-cultured embryonic primary neurons carrying the same combinations of mutant alleles in homozygosis (indicated on the left; used alleles: tub84Bdef, mspsA, tauKO/Df, eb104524, staiKO). D,E) Plotting comet velocity or lifetime against Eb1 amounts from different genetic conditions shows good correlation (r and p-value determined via non-parametric Spearman correlation analysis). For raw data see S8 Data. (TIF) [file pgen.1009647.s002.tif]

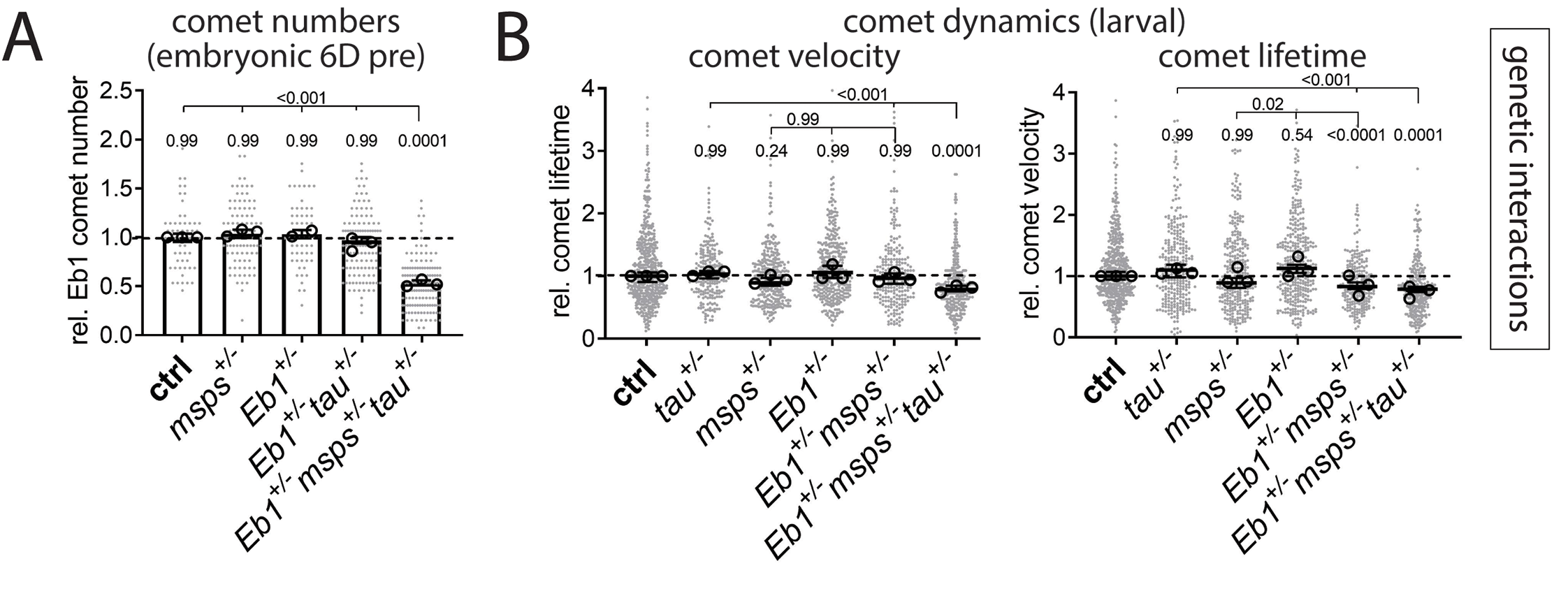

Supplement: S3 Fig — A) Eb1 comet numbers in fixed primary neurons cultured for 12hrs following 5 day pre-culture. B) Comet velocity and lifetime obtained from live analyses of primary neurons cultured for 18hrs from CNSs of late larvae carrying single-, double- or triple-heterozygous conditions (complementing data in Fig 2A). In all graphs, data were normalised to parallel controls (dashed horizontal lines) and are shown as scatter dot plots with median ± 95% confidence interval (B) or bar chart with mean ± SEM (A) of at least two experimental repeats; large open circles in graphs indicate median/mean of independent biological repeats. P-values obtained with Kruskal-Wallis ANOVA test are given above data points/bars; used alleles: mspsA, tauKO, Eb104524. For raw data see S9 Data. (TIF) [file pgen.1009647.s003.tif]

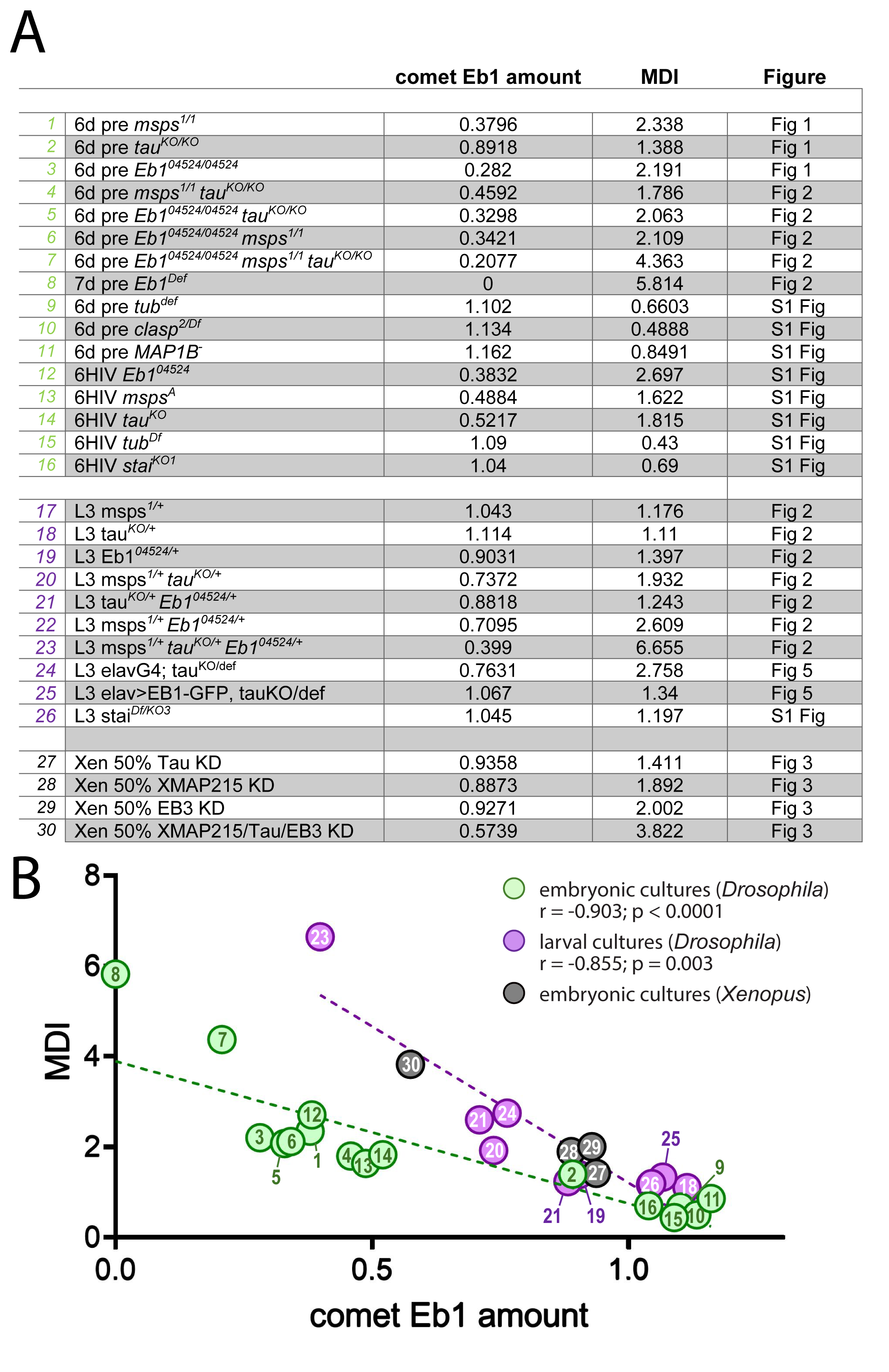

Supplement: S4 Fig — The data and graph show further details behind the correlations displayed in Fig 2C. A) The table provides descriptions, data and references for the graph shown in B: coloured numbers (1st column) correspond to numbers of data points in B; the different allelic combinations and culture conditions used (2nd column) comprise embryonic neurons cultures for 6 hrs (6HIV), embryonic neurons cultured for 12 hrs following 6 day preculture (6d pre), neurons cultured from larval CNSs for 18hrs (L3); respective data for Eb1 amounts (3rd column) and MT curling (4th column) were obtained from different sets of experiments throughout this work (5th column lists the figures from where these data originate). B) Correlation plot of the data shown in A, with numbers and colours of data points corresponding to the 1st column; r and p-value determined via non-parametric Spearman correlation analysis. For raw data see S10 Data. (TIF) [file pgen.1009647.s004.tif]

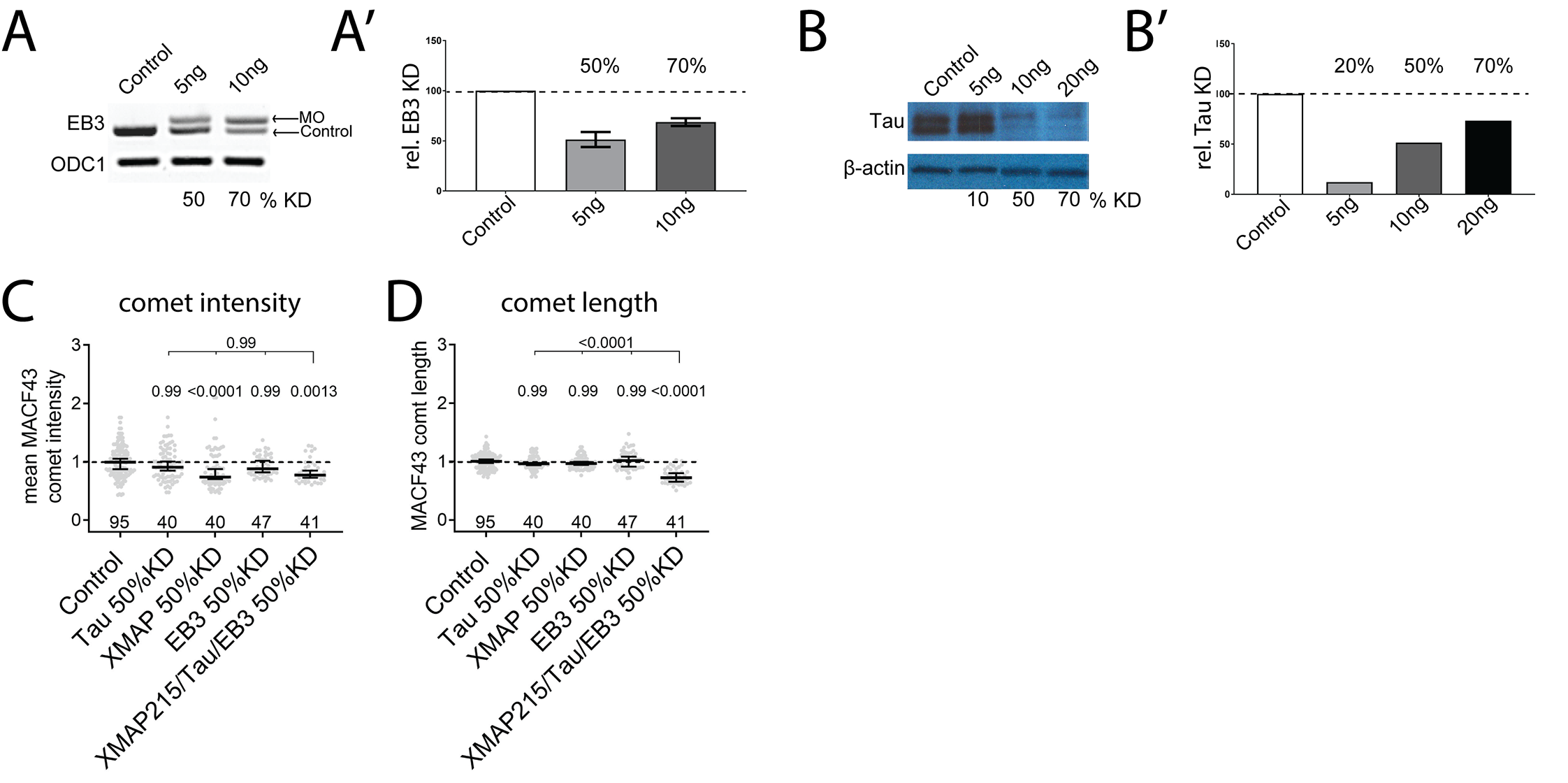

Supplement: S5 Fig — A-B’) A RT-PCR DNA gel (A) and Western blot (B) and their quantifications (A’, B’) show the degrees of EB3/Tau knock-downs upon application of different morpholino concentrations (indicated on top in blots and at the bottom in graphs); ODC1 and ß-actin are used as loading controls; data are normalised to no-morpholino controls from two experimental repeats (dashed lines). 50% knock-down of XMAP215 was achieved by injecting 6 ng of the validated XMAP215 MO as described previously [47,56]. C-D) Different properties of MACF43::GFP comets (as indicated upon graphs) obtained from Xenopus primary neurons, either upon 50%; data were normalised to parallel controls (dashed horizontal lines) and are shown as median ± 95% confidence interval; merged sample numbers from at least two experimental repeats are shown at the bottom, P-values obtained with Kruskall-Wallis ANOVA and Dunn’s posthoc tests above data points. For raw data see S11 Data. (TIF) [file pgen.1009647.s005.tif]

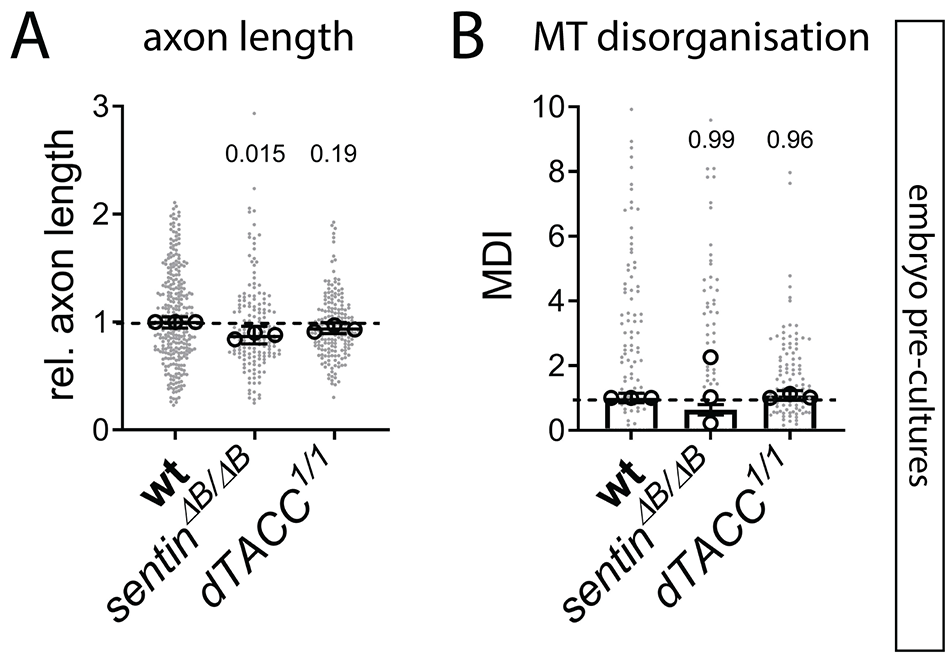

Supplement: S6 Fig — Axon length (A) and MT curling (B) embryonic 6d pre-cultured neurons which were either wild-type (wt) or homozygous mutant for sentin or dTACC (as indicated); data were normalised to parallel controls (dashed horizontal lines) and are shown as scatter dot plots with median ± 95% confidence interval (A) or mean ± SEM (B) from at least two experimental repeats; large open circles in graphs indicate median/mean of independent biological repeats. P-values obtained with Kruskal-Wallis ANOVA tests are shown above data points/bars. For raw data see S12 Data. (TIF) [file pgen.1009647.s006.tif]

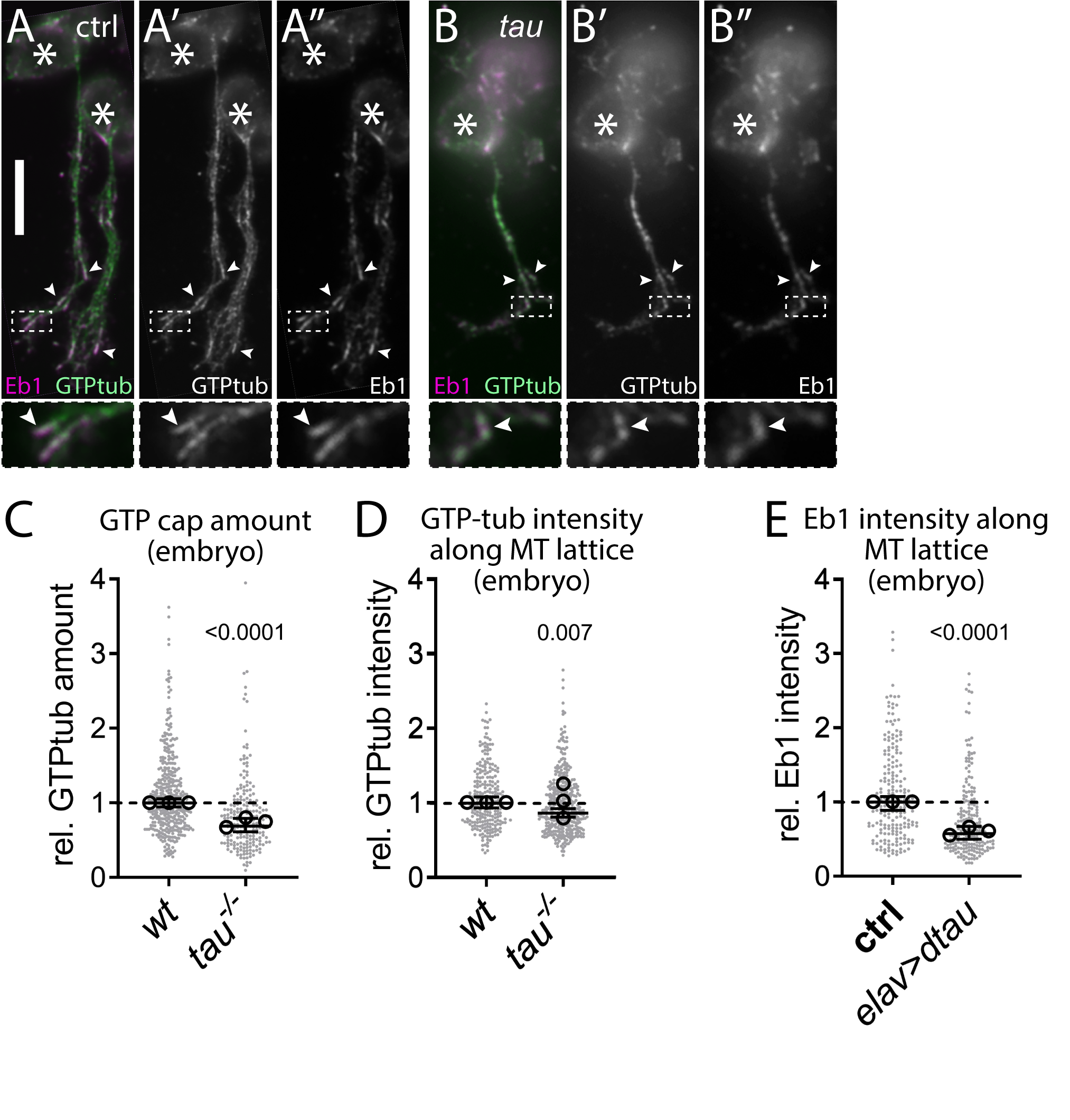

Supplement: S7 Fig — A-B”) Fixed primary neurons stained for Eb1 (magenta) and GTP-tubulin (green; the scale bar represents 10μm); asterisks indicate somata, dashed boxes the positions of the 4-fold magnified close-ups shown at the bottom, arrowheads point at Eb1::GFP comets and GTP caps. C,D) Graphs showing staining intensity of GTP-tubulin at MT plus-ends (C) and along the MT lattice (D) of embryonic neuron at 6 HIV. E) Graphs showing staining intensity of Eb1 along the MT lattice of neurons without/with elav-Gal4-driven expression of Drosophila Tau (dtau). Overexpression of dtau leads to a reduction of Eb1 at the MT shaft; data were normalised to parallel controls (dashed horizontal lines) and are shown as scatter dot plots with median ± 95% confidence interval from at least two independent repeats with 3 experimental replicates; large open circles in graphs indicate median/mean of independent biological repeats. P-values obtained with Kruskall-Wallis ANOVA test above data points/bars. For raw data see S13 Data. (TIF) [file pgen.1009647.s007.tif]

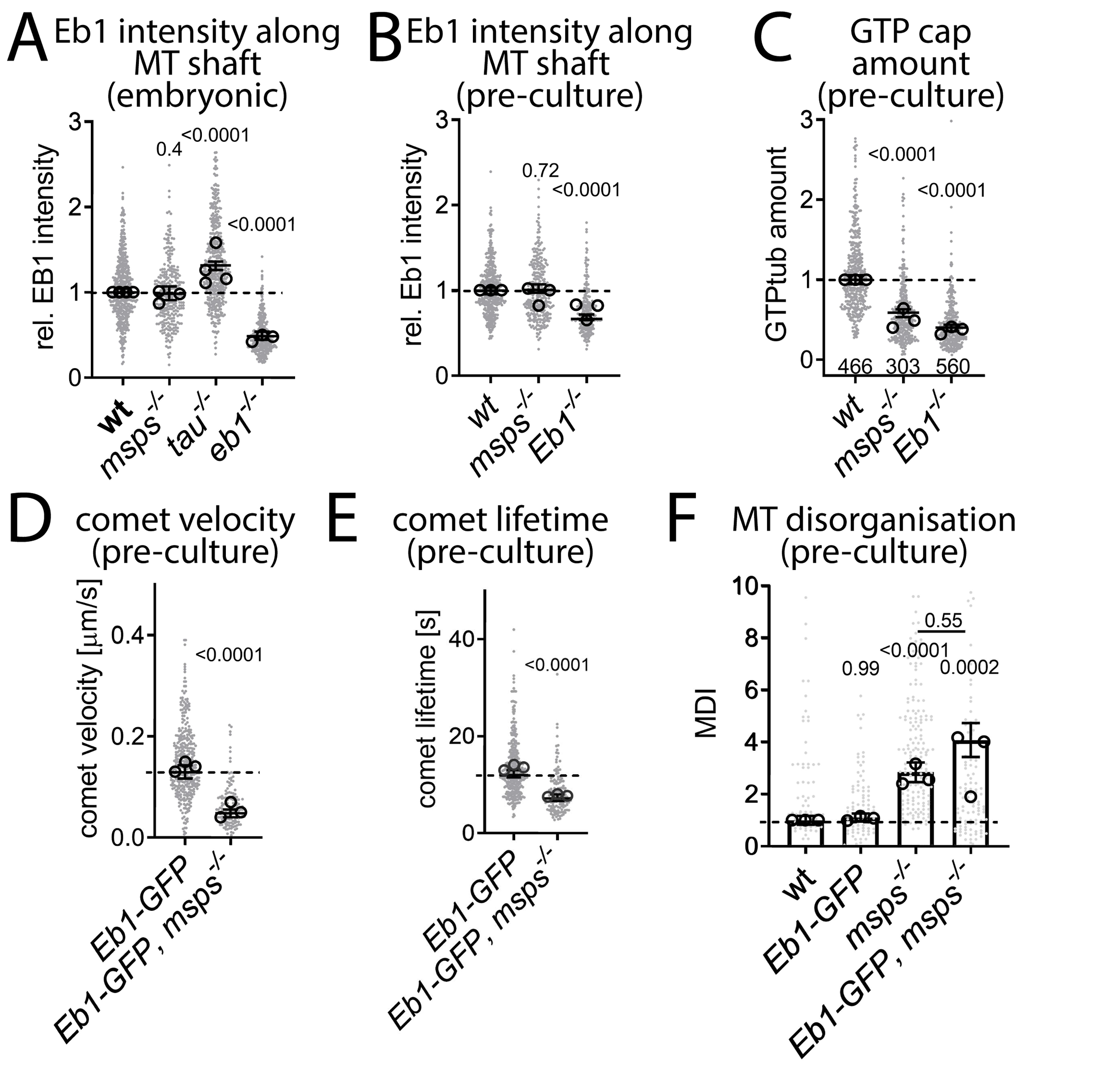

Supplement: S8 Fig — A,B) Eb1 shaft localisation is unaffected by loss of Msps in primary neurons at 6HIV (A) or at ~12HIV following 6 day pre-culture (B). C) Staining intensity of GTP-tubulin at MT plus-ends is reduced in mspsA/A and Eb104524/04524 mutant embryonic neurons at ~12 HIV following 6 day pre-culture. D-F) Expressing Eb1::GFP via elav-Gal4 does not improve comet velocities (D), comet lifetime (E) and MT curling (F) in primary neurons of mspsA/146 mutants (cultured12 HIV following 6 day pre-culture); data were normalised to parallel controls (dashed horizontal lines) and are shown as scatter dot plots with mean ± SEM (F) or median ± 95% confidence interval (A-E) from at least two experimental repeats; large open circles in graphs indicate median/mean of independent biological repeats. P-values obtained with Kruskal-Wallis ANOVA tests and Dunn’s posthoc analyses are shown above data points/bars. For raw data see S14 Data. (TIF) [file pgen.1009647.s008.tif]

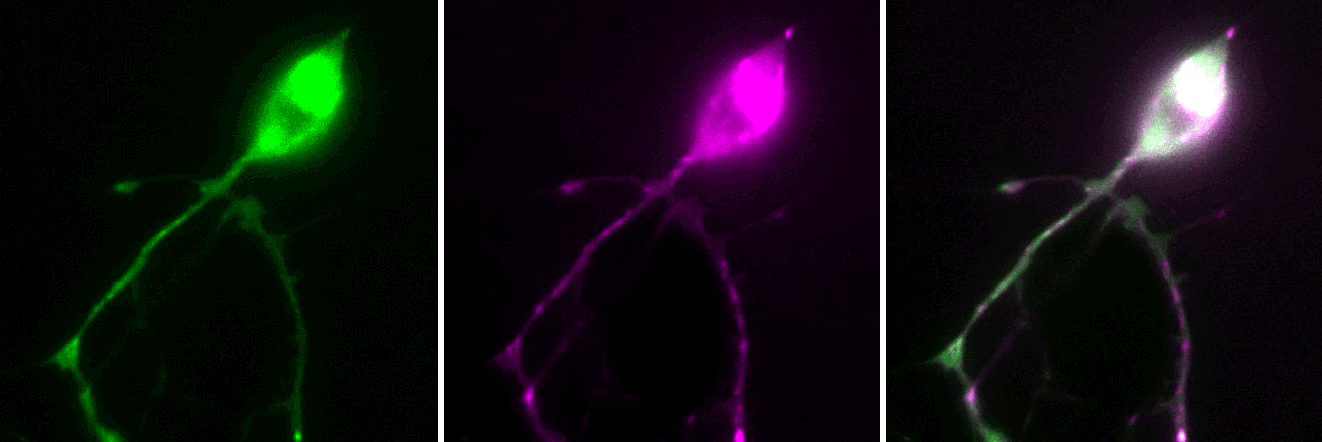

Supplement: S1 Movie — Live movie of a wild-type neuron co-expressing Msps::GFP and Eb1::RFP; for stills see Fig 4A–4A”. As indicated, single channels are shown on the left and middle, and the combined movie on the right. The movie was acquired at 0.5 frames per second and plays at 0.5 s per frame. The scale bar indicates 10 μm. (GIF) [file pgen.1009647.s023.gif]

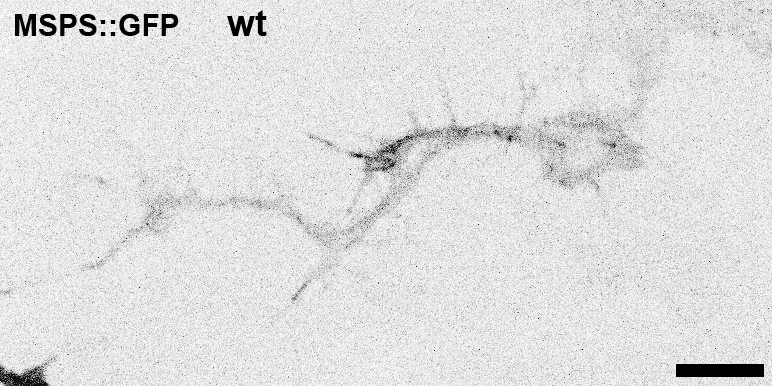

Supplement: S2 Movie — Live movie of a wild-type neuron expressing Msps::GFP; for a still see Fig 4C. The movie was acquired at 1 frame per second and plays at 0.2 s per frame. The scale bar indicates 10 μm. (GIF) [file pgen.1009647.s024.gif]

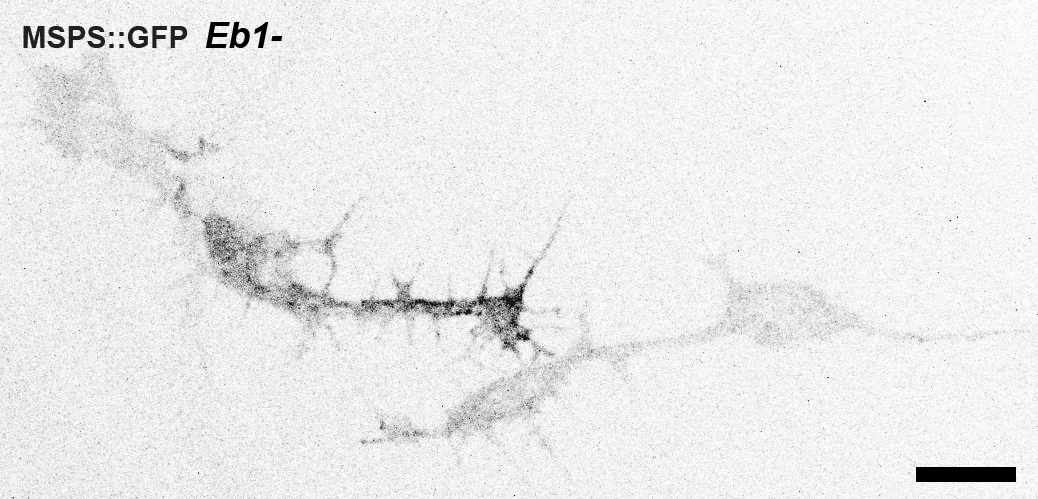

Supplement: S3 Movie — Live movie of an Eb104524/04524 mutant neuron expressing Msps::GFP; for a still see Fig 4C’. The movie was acquired at 1 frames per second and plays at 0.2 s per frame. The scale bar indicates 10 μm. (GIF) [file pgen.1009647.s025.gif]
