# Supplementary material for: Tau, XMAP215/Msps and Eb1 co-operate interdependently to regulate microtubule polymerisation and bundle formation in axons
Source: PLoS Genet. 2021 Jul 6;17(7):e1009647. doi: 10.1371/journal.pgen.1009647 (PMC8284659; doi:10.1371/journal.pgen.1009647)
Supplement: Attachment — Submitted filename: response to reviewers.docx [file pgen.1009647.s026.docx]

The function of Msps in microtubule polymerization was analyzed extensively in dividing cells in which centrosomes serve as the microtubule organizing center. In mature neurons, centrosomes are immature and acentrosomal microtubules are organized in both axons and dendrites. It would worth a discussion to emphasize the novelty of this study in revealing the role of Msps in organizing acentrosomal microtubules for axonal transport. It would also be good to cite the following three papers on the role of Msps on acentrosomal microtubule regulation in various cell types.

Quan Tang et al., EMBO J 2020;

Yiming Zheng et al., Nature Cell Biol. 2020;

Qiannan Deng et al., BioRxiv 2020.

Tang et al. 20 was cited already, the Zheng and Deng references were inserted in the last Discussion section in the context of XMAP215 as a promoter of nucleation, which would mean acentrosomal nucleation in axons.

“Rescue” needs to be used more accurately. When introducing a different gene to a mutant/RNAi condition, “suppression” should be used formally, as “rescue” often refers to the result by reintroducing the same gene that was mutated/depleted in the mutant/RNAi.

Has been corrected throughout the text.

The fact that variants in CKAP5/XMAP215 and EB1 have not been identified in human patients

with neurological disorders might be due to the essential function of CKAP5 and/or EB1 during

embryonic development. The author might want to consider this possibility when discussing the

disease implications.

This point was added together with a reference discussing this issue in greater detail.

Define HIV in the first place.

The explanation has been inserted in the first result section.

The references on line 81 need to be formatted.

Formatting has been mended.

1) The results with EB1 facilitating msps recruitment without the necessity of other adaptors is very interesting and the overall the result is very nicely discussed and placed in the context of the current literature in the field. The authors clearly show that the CTD is dispensible, indicating that adaptors are not necessary to recruit msps to MT plus-ends in fly neurons. It would be nice for the authors to discuss these results a bit in the discussion (page 13 or 14), to substantiate their claim that there is not a direct interaction between msps and eb1 (or any other adaptor), but a through lattice facilitation of the binding of one another.

These adaptors are being dealt with in the second Discussion section.

2) An interesting experiment/control would be to analyze EB1 intensity on the microtubule lattice upon overexpression or knockdown of DCX-EMAP, especially since the binding sites are more similar. This may prove to be difficult depending on which fly lines are available and is not necessary for the publication of this paper, but it would be interesting if DCX were playing a similar or different role with regards to effects on EB1.

We would love to do this experiment but obtaining and rebalancing the flies would cause an unnecessary delay of this revision. We therefore decided to omit it.

3) In the last paragraph of the tau discussion on page 14, tau has also been shown to prevent MAP7 from binding the same region of the lattice (Monroy et al., Nat Comm, 2018), similarly to how it prevents MAP6 and now EB1. Citing this paper would help support the more global role for tau in this function. In addition, the authors should cite the work on tau by Tan et al., NCB, 2019 showing that tau forms oligomeric assemblies on the microtubule that are capable of blocking other MAPs, motors, and severing enzymes. This shows a clear mechanism by which tau is able to dictate access of other proteins to the microtubule lattice, lending further support for the observations in this paper.

Thanks for heads-up. This reference is now included and MAP7 mentioned.

4) There are some minor typos throughout, such as Fig 2: MT disorganisaiton should be “disorganisation”.

We carefully re-read the manuscript and made minor corrections throughout, including reformulating some sentences to make them clearer.

FURTHER CHANGES:

We carefully checked over the methods part and reshuffled some parts into more appropriate positions. Also in the 3^rd^ paragraph we reformulated the statement about Tau adding some references detailing the problems associated with dominant links to disease, which often do not reflect the intrinsic function of these genes. We felt this was not well explained in the original version of the manuscript.
